# Supplementary material for: Comparison of ERlangen Score with pTau/Aβ1-42 Ratio for Predicting Cognitive Decline and Conversion to Alzheimer’s Disease
Source: Brain Sci. 2025 Mar 23;15(4):334. doi: 10.3390/brainsci15040334 (PMC12026031; doi:10.3390/brainsci15040334)
Supplement: Supplementary file 1 [file brainsci-15-00334-s001.zip › brainsci-3496674-supplementary.pdf]

Supplementary Material

Table S1. Optimal cut-off values of the pTau/Aβ1-42 ratio for different immunoassay combinations.

	n	Cut-off
Innotest® Innogenetics[1]	204	0.091
Innotest® Fujirebio[1]	169	0.079
Lumipulse® Fujirebio	172	0.067

n = the number of neurochemically definite AD cases and biomarker-negative controls was employed in the calculation using the Youden index. 1 = Innotest® was used for the measurement of pTau181. Aβ1-42 was measured using IBL immunoassays in both cases.

Table S2. Baseline characteristics of patients classified according to ERS categories (0-4).

	0		1		2		3		4	
	n	**%**	***n***	**%**	***n***	**%**	***n***	**%**	***n***	**%**
Total (female)	107 40	41.3 38.5	49 16	18.9 15.4	4 1	1.5 1.0	63 31	24.3 29.8	36 16	13.9 15.4
MCI (female)	82 30	39.4 35.3	35 10	16.8 11.8	2 1	1.0 1.2	56 29	26.9 34.1	33 15	15.9 17.6
SCI (female)	25 10	49.0 52.6	14 6	27.5 31.6	2 0	3.9 0.0	7 2	13.7 10.5	3 1	5.9 5.3
	mean	***SEM***	***mean***	***SEM***	***mean***	***SEM***	***mean***	***SEM***	***mean***	***SEM***
Age [years]	62	0.8	65	1.2	72	4.4	72	1.0	74	1.4
Age [years] in MCI group	63	0.9	66	1.4	79	2.0	71	1.0	74	1.5
Age [years] in SCI group	60	1.6	64	1.0	64	1.0	75	2.5	64	4.6
Education [years] in MCI group	14	0.3	14	0.4	15	1.0	13	0.4	13	0.6
Education [years] in MCI group	14	0.4	14	0.5	16	1.0	13	0.5	13	0.6
Education [years] in SCI group	14	0.6	13	0.7	13	1.0	15	1.4	15	2.7
MMSE	28	0.2	27	0.5	26	0.2	27	0.5	27	0.5
MMSE in MCI group	27	0.2	26	0.4	26	0.4	26	0.3	26	0.3
MMSE in SCI group	29	0.2	29	0.3	28	0.0	28	0.3	28	0.6
Follow-Up [years]	4.5	0.4	4.7	0.6	6.2	1.0	3.6	0.3	2.9	0.3
Follow-Up [years] in MCI group	4.3	0.4	4.1	0.6	6.9	0.3	3.3	0.3	2.9	0.3
pTau181 [pg/ml]1	32.3	2.3	66.5	2.8	N/A	N/A	97.2	15.6	82.1	14.1
pTau181 [pg/ml]2	39.5	2.4	72.7	3.6	N/A	N/A	117.9	11.2	92.1	8.2
pTau181 [pg/ml]3	40.0	1.4	62.2	3.5	52.9	7.6	106.8	11.3	91.1	7.0
Aβ1-42 [pg/ml]4	986	42	1379	67	N/A	N/A	674	29	495	28
Aβ1-42 [pg/ml]3	1006	53	1302	117	923	180	742	28	514	31
Aβ1-42/ Aβ1-40 ratio 4	0.087	0.002	0.075	0.003	N/A	N/A	0.040	0.001	0.039	0.002
Aβ1-42/ Aβ1-40 ratio 3	0.083	0.002	0.075	0.004	0.044	0.005	0.042	0.002	0.032	0.003

2 = Innotest® Fujirebio

3 = Lumipulse® Fujirebio

4 = IBL® immunoassay

SEM = standard error of mean, 1 = Innotest® Innogenetics, 2 = Innotest® Fujirebio, 3 = Lumipulse® Fujirebio, 4 = IBL® immunoassay

Table S3. Baseline characteristics of individuals classified according to the different immonuassays used.

	Innotest® Innogenetics		Innotest® Fujirebio		Lumipulse® Fujirebio	
	n		***n***		***n***	
Total (female)	103 (45)		99 (27)		57 (22)	
Conversion to dementia	34		28		13	
	mean	***SEM***	***mean***	***SEM***	***mean***	***SEM***
Age [years]	67	1.0	65	0.9	69	8.8
Education [years]	14	0.3	14	0.3	14	3.1
MMSE	27	0.2	27	0.2	26	1.9
Follow-Up [years]	3.4	0.2	6.7	0.4	1.4	0.1

Table S4. The root mean squared error of the predicted fixed effects (Marginal RMSE) and the fixed and random effects (Conditional RMSE) are demonstrated in order to evaluate the accuracy of the linear mixed models for the groups with either the ordinally scaled ERS, the binary ERS, the groups defined by the pTau/Aß1-42 ratio, and the ERS without the Aß1-42/Aß1-40 ratio.

	**Marginal RMSE**	**Conditional RMSE**
ERS	1.668	0.949
ERS binary	1.673	0.949
pTau/Aß1-42 ratio	1.698	0.943
ERS w/o Aß1-42/Aß1-40 ratio	1.798	0.948

Table S5. Linear mixed model model fits and estimates for comparison of MMSE z scores and their trajectories between normal and pathological ERS scores (A) and pTau/Ab42 ratio (B) for SCI (1) and MCI Groups (2).

**1A**	Fixed effects					
	**Estimate**	***SEM***	***t***	***p***	**95% CI**	
Intercept	-0.46	0.14	-3.289	0.002	-0.74	-0.18
FollowUp [Y]	-0.07	0.03	-2.032	0.046	-0.14	0.00
pathologic ERS	-0.5	0.28	-1.787	0.079	-1.06	0.06
pathologic ERS x FollowUP [Y]	-0.06	0.08	-0.767	0.446	-0.22	0.10
**Model fit**						
AIC	491.557					
BIC	537.510					
Marginal RMSE	1.073					

**1B**	Fixed effects					
	**Estimate**	***SEM***	***t***	***p***	**95% CI**	
Intercept	-0.55	0.14	-3.952	< .001	-0.83	-0.27
FollowUp [Y]	-0.08	0.04	-1.972	0.050	-0.15	0.00
pathologic pTau / Aβ42 ratio	-0.13	0.29	-0.445	0.658	-0.71	0.45
pathologic pTau / Aβ42 ratio x FollowUP [Y]	-0.05	0.09	-0.578	0.564	-0.23	0.12
**Model fit**						
AIC	519.162					
BIC	545.954					
Marginal RMSE	1.088					

**2A**	Fixed effects					
	**Estimate**	***SEM***	***t***	***p***	**95% CI**	
Intercept	-1.6	0.1	-16.431	< .001	-1.78	-1.4
FollowUp [Y]	-0.1	0.06	-1.694	0.103	-0.21	0.02
pathologic ERS	-0.17	0.16	-1.072	0.285	-0.48	0.14
pathologic ERS x FollowUP [Y]	-0.52	0.09	-5.461	< .001	-0.71	-0.32
**Model fit**						
AIC	1.784.749					
BIC	1.822.644					
Marginal RMSE	1.679					

**2B**	Fixed effects					
	**Estimate**	***SEM***	***t***	***p***	**95% CI**	
Intercept	-1.59	0.09	-16.822	< .001	-1.78	-1.4
FollowUp [Y]	-0.11	0.05	-2.182	0.039	-0.23	-0.01
pathologic pTau / Aβ42 ratio	-0.17	0.16	-1.054	0.294	-0.48	0.14
pathologic pTau / Aβ42 ratio x FollowUP [Y]	-0.54	0.1	-5.386	< .001	-0.73	-0.33
**Model fit**						
AIC	1.767.367					
BIC	1.823.262					
Marginal RMSE	1.684					

Table S6. Comparison of Area Under the Curve (AUC) of Receiver Operating Characteristic (ROC) Curves for predicting dementia risk by pTau/Aβ1-42 Ratio and ERS for the Different Immunoassays used.

	pTau/Aβ1-42 ratio	ERS	p	AUC-Difference	95% CI	
Innotest® Innogenetics[1]	.752	.731	.497	.021	- .039	.081
Innotest® Fujirebio[1]	.768	.740	.247	.028	- .019	.076
Lumipulse® Fujirebio	.645	.620	.559	.025	- .060	.111

Table S7. Sensitivity, specificity, positive and negative likelihood ratios of binary classifications of pTau/Aβ1-42 Ratio and ERS.

	pTau/Aβ42 ratio		ERS	
		95% CI		95% CI
Sensitivity	0.65	[0.54, 0.76]	0.68	[0.57, 0.78]
Specifitiy	0.72	[0.65, 0,79]	0.73	[0.66, 0.79]
LR+	2.38	[1.79, 3,16]	2.52	[1.89, 3.35]
LR-	0.48	[0.35, 0.65]	0.44	[0.32, 0.61]

1 95% CI is shown in brackets

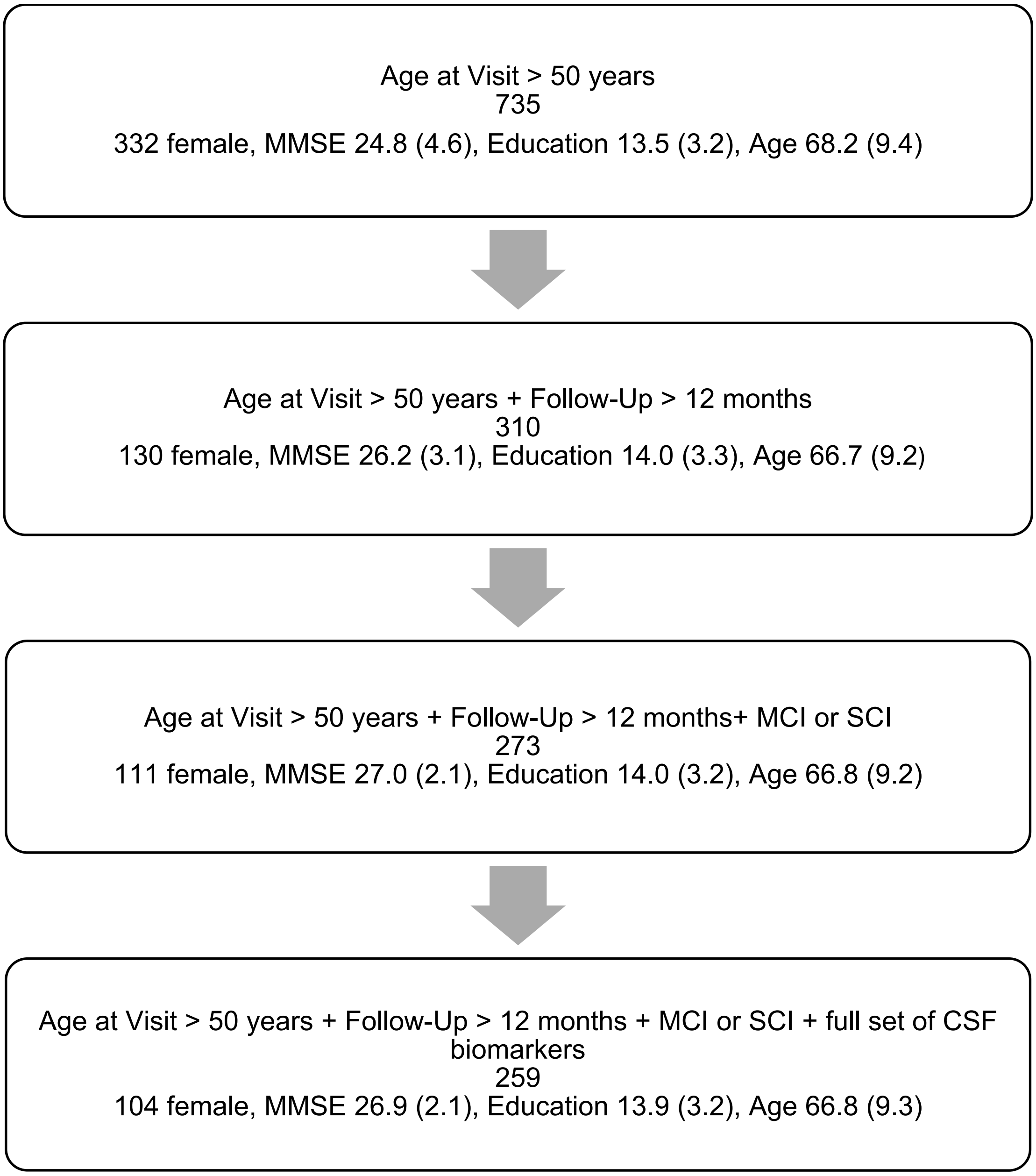

Figure S1. Participant Flowchart. Out of a total of 753 patients, 259 were eligible to participate in our study. MCI = mild cognitive impairment; SCI = subjective cognitive impairment; the figures in brackets indicate the standard deviation.

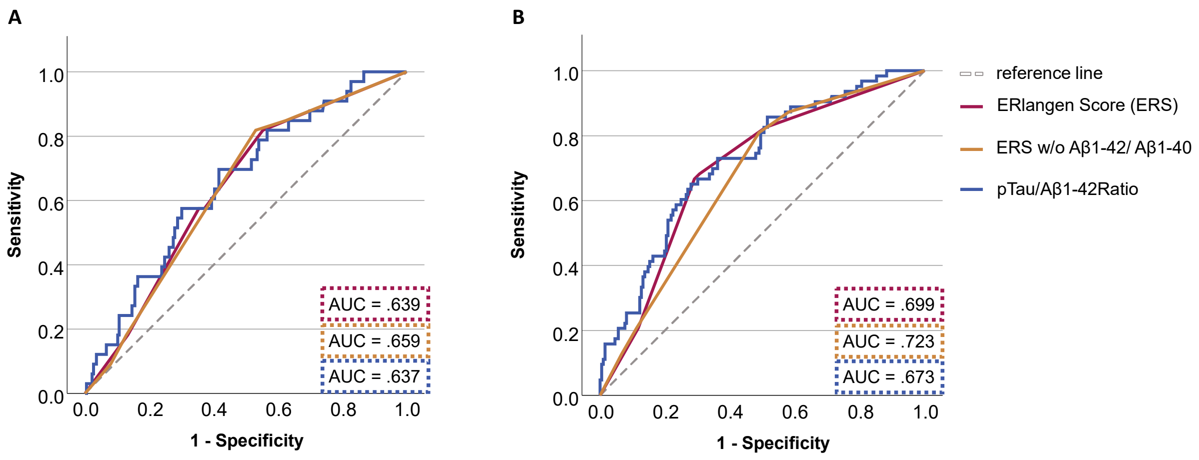

Figure S2. The receiver operating characteristic (ROC) curve is utilised for the prediction of dementia, employing the Erlangen score (ERS), the pTau/Aβ42 ratio, and the ERS excluding the Aβ1-42/Aβ1-40 ratio. The hazard probabilities are predicted from a Cox regression model, which was specified for a 2-year follow-up period (A) and a 5-year follow-up period (B). AUC = area under the curve
